# Crystal structure of bis­(tetra­methyl­thio­urea-κ*S*)bis(thio­cyanato-κ*N*)cobalt(II)

**DOI:** 10.1107/S205698902001021X

**Published:** 2020-07-31

**Authors:** Aleksej Jochim, Rastko Radulovic, Inke Jess, Christian Näther

**Affiliations:** a Institut für Anorganische Chemie, Christian-Albrechts-Universität Kiel, Max-Eyth Str. 2, D-24118 Kiel, Germany

**Keywords:** crystal structure, cobalt thio­cyanate, tetra­methyl­thio­urea, discrete complexes, thermal properties

## Abstract

In the course of systematic investigations on the synthesis of Co(NCS)_2_ coordination compounds with different thio­urea ligands, the title compound, [Co(NCS)_2_(C_5_H_12_N_2_S)_2_], was obtained. In this compound the Co^II^ cations are coordinated by two crystallographically independent N-bonded thio­cyanate anions and two tetra­methyl­thio­urea ligands into discrete complexes that are located in general positions and show a strongly distorted tetra­hedral geometry. Inter­molecular C—H⋯S hydrogen bonds of different strength can be observed between the discrete complexes, which are connected by pairs of hydrogen bonds into zigzag-like chains that elongate in the *b*-axis direction. These chains are additionally linked by strong C—H⋯S hydrogen bonds along the *a*-axis direction, resulting in the formation of layers that are parallel to the *ab* plane. There is also one weak intra­molecular C—H⋯S hydrogen bond between two neighbouring thio­urea ligands within the complexes. Comparison of the experimental PXRD pattern with that calculated from the single-crystal data prove that a pure phase has been obtained. Thermoanalytical investigations reveal that this compound melts at 364 K and decomposes upon further heating.

## Abstract 2

The title compound consists of discrete complexes with the composition Co(NCS)_2_(tetra­methyl­thio­urea)_2_, in which the Co^II^ cations are tetra­hedrally coordinated by two N-bonded thio­cyanate anions and two tetra­methyl­thio­urea ligands. These complexes are linked by inter­molecular C—H⋯S hydrogen bonds into layers that are parallel to the *ab* plane.

## Chemical context   

The thio­cyanate anion is a very versatile ligand, which can coordinate in many different ways to metal cations, leading to compounds with a variety of coordination networks (Buckingham, 1994 ▸; Haasnoot *et al.*, 1984 ▸; Barnett *et al.*, 2002 ▸; Bhowmik *et al.*, 2010 ▸; Abedi *et al.*, 2016 ▸). This ligand is also able to mediate reasonable magnetic exchange (Palion-Gazda *et al.*, 2015 ▸), which is one reason why we have been inter­ested in transition-metal thio­cyanate coordination compounds for many years. In this context, we are especially inter­ested in compounds of the general composition [*M*(NCS)_2_(*L*)_2_]_*n*_, in which paramagnetic first-row transition-metal cations *M* such as Mn^II^, Fe^II^, Co^II^ or Ni^II^ are octa­hedrally coordinated by two N- and two S-bonding thio­cyanate anions and two coligands *L* that usually consist of pyridine derivatives. Depending on the nature of the coligand, the metal cations are connected into chains by pairs of μ-1,3 coordinating anionic ligands (Mautner *et al.*, 2018 ▸; Prananto *et al.*, 2017 ▸; Shurdha *et al.*, 2013 ▸; Jin *et al.*, 2007 ▸; Böhme *et al.*, 2020 ▸), or they are linked into layers with different layer topologies (Werner *et al.*, 2015*a* ▸; Neumann *et al.*, 2018*a* ▸; Suckert *et al.*, 2016 ▸). The chain compounds show either ferromagnetism (Neumann *et al.*, 2019 ▸), anti­ferromagnetism (Jochim *et al.*, 2020 ▸) or they represent anti­ferromagnetic phases of single-chain magnets (Mautner *et al.*, 2018 ▸; Rams *et al.*, 2017 ▸, 2020 ▸; Werner *et al.*, 2015*b* ▸), whereas the layer compounds are in most cases ferromagnets (Suckert *et al.*, 2016 ▸). For this composition a third structure type is known, in which the metal cations are tetra­hedrally coordin­ated, forming discrete complexes with only N-terminally bonded thio­cyanate anions (Neumann *et al.*, 2018*b* ▸). For some coligands, at least two of the three isomers can be obtained. With 4-acetyl­pyridine as coligand, for example, the chain as well as the layer isomer can be prepared, with the latter representing the thermodynamically stable form at room temperature (Werner *et al.*, 2015*a* ▸). If 4-meth­oxy­pyridine is used as coligand, the chain isomer as well as the tetra­hedral discrete complex can be obtained, and in this case the chain compound is thermodynamically stable at room temperature (Mautner *et al.*, 2018 ▸; Rams *et al.*, 2020 ▸). Finally, different polymorphic modifications can also be obtained for discrete complexes (Neumann *et al.*, 2018*b* ▸).
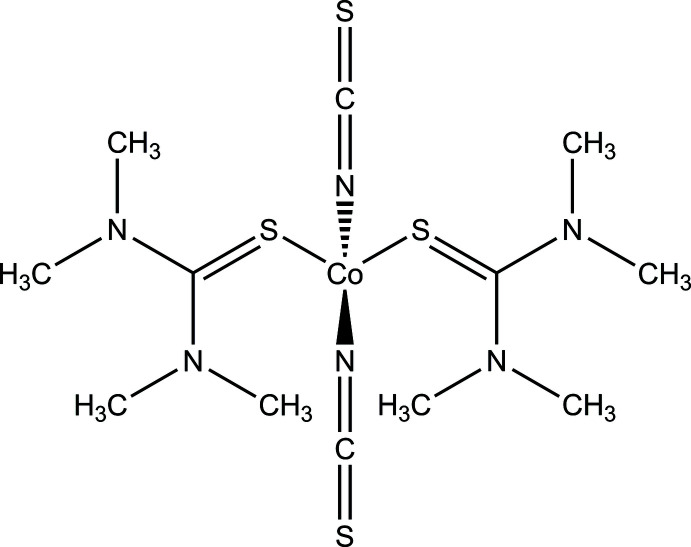


However, in all previous work we exclusively used N-donor coligands for the synthesis of Co(NCS)_2_ thio­cyanate coordin­ation polymers, and to investigate the influence of the coligand on the structure and the magnetic behaviour, we became inter­ested in donor ligands that can coordinate *via* a sulfur atom, including thio­urea derivatives. With thio­urea, the crystal structure of one Co(NCS)_2_ coordination compound has already been already reported, and in this case the Co cations are linked by pairs of thio­urea sulfur atoms, whereas the thio­cyanate anions are only terminally N-bonded (Rajarajan *et al.*, 2012 ▸). Independent of this, we used ethyl­ene­thio­urea as coligand and obtained a compound with the composition [Co(NCS)_2_(ethyl­ene­thio­urea)_2_]_*n*_. Single-crystal structure determination proves that, in this case, the Co^II^ cations are connected by pairs of thio­cyanate ligands into chains, which corresponds exactly to the desired structure (Jochim *et al.*, 2020 ▸). In contrast to the analogous compounds with N-donor coligands, this compound shows anti­ferromagnetic ordering but no relaxations of single chains. To investigate this in more detail, we used tetra­methyl­thio­urea as coligand and obtained crystals of the title compound Co(NCS)_2_(tetra­methyl­thio­urea)_2_. Surprisingly, this compound consists of discrete complexes, in which the Co^II^ cations are tetra­hedrally coord­inated, which is also reflected in its IR spectra, where the C—N stretching vibration of the thio­cyanate anion is observed at 2048 cm^−1^ (see Fig. S1 in the supporting information). The powder diffraction pattern reveals that a pure product has been obtained (Fig. S2). The thermogravimetric (TG) curve shows that all tetra­methyl­thio­urea ligands are emitted in one step (theoretical mass loss: 60.2%), which is accompanied by an exothermic peak in the differential thermoanalysis (DTA) curve (Fig. S3). However, two endothermic peaks are observed at low temperatures where the sample mass does not change. To investigate this phenomenon in more detail, measurements using differential scanning calorimetry (DSC) were performed, which prove that the first endothermic signal is reversible with some hysteresis, pointing to some structural transition (Fig. S4), which could explain why no changes in the PXRD pattern are observed after cooling. The second endothermic peak is irreversible, but thermomicroscopic measurements prove that this event corresponds to melting, which means that upon cooling no crystallization is observed (Figs. S5 and S6).

## Structural commentary   

**Figure 1:**
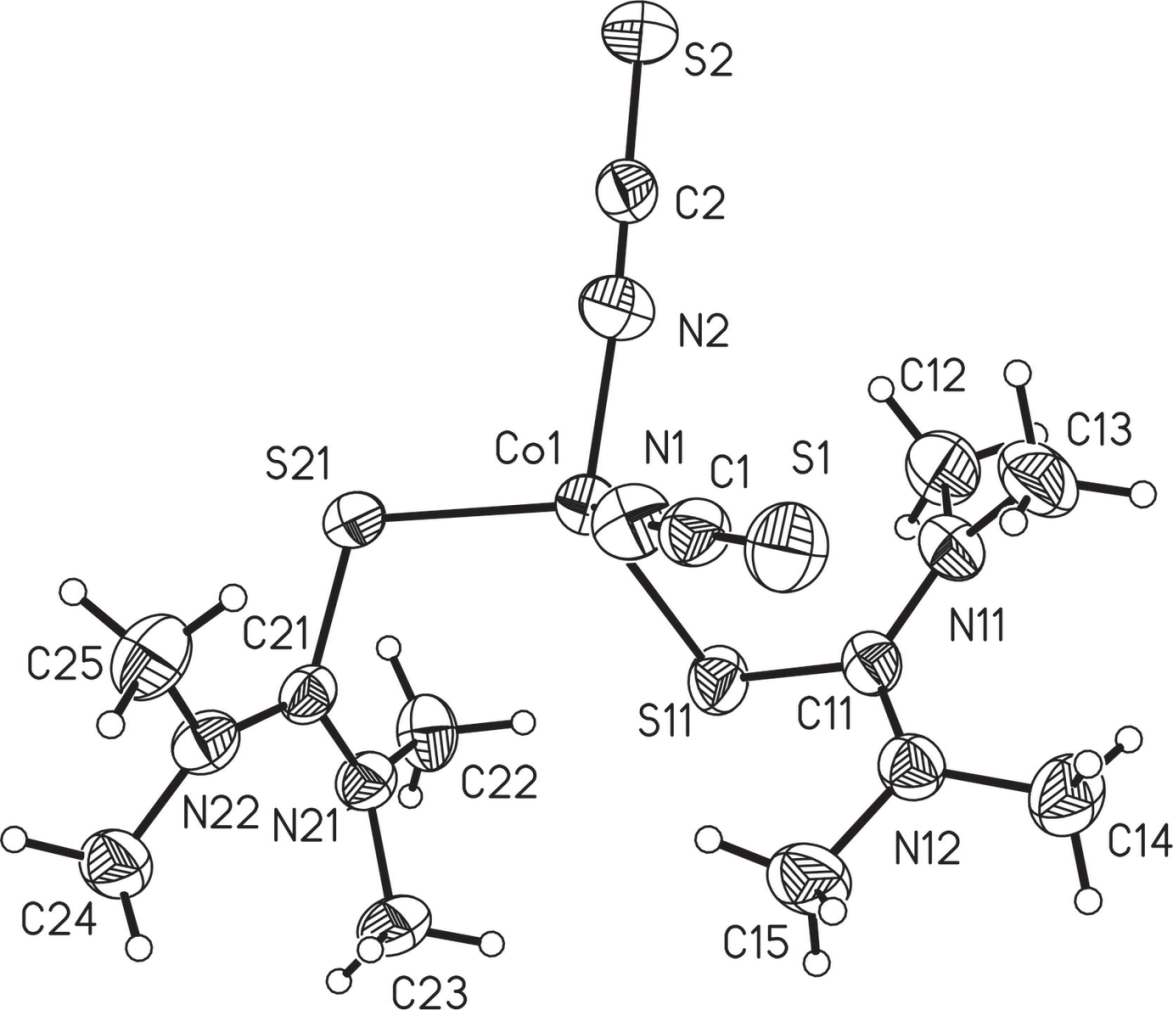
View of the asymmetric unit of the title compound with the atom labelling and displacement ellipsoids drawn at the 50% probability level.

The asymmetric unit of the title compound contains two crystallographically independent tetra­methyl­thio­urea mol­ecules, two thio­cyanate anions and one Co^II^ cation in general positions (Fig. 1 ▸). The Co^II^ cations are coordinated by two N-bonded thio­cyanate anions and two tetra­methyl­thio­urea mol­ecules into discrete complexes, with bonds lengths and angles similar to those reported in the literature (Table 1 ▸). The coordination polyhedra around the Co^II^ cations can be described as strongly distorted tetra­hedra (Table 1 ▸), which is also obvious from the tetra­hedral angle variance σ_θ〈tet〉_^2^ = 81.0 and the mean tetra­hedral quadratic elongation 〈λ_tet_〉 = 1.036 (Robinson *et al.*, 1971 ▸). The C—N bond lengths between the thio­ketone C and the amino groups are significantly shorter than those between the amino groups and the methyl C atoms, which points to some degree of double-bond character of the former. This is expected as thio­ketones are subject to thio­ketone–enthiole tautomerism similar to the tautomerism found for regular ketones, which is also supported by the fact that the CNMe_2_ groups are planar with angles close to 120° (Devillanova, 2007 ▸). The NMe_2_ groups of the same coligand are twisted against each other with angles of 45.74 (9) and 46.32 (8)° for the two crystallographically independent tetra­methyl­thio­urea coligands.

## Supra­molecular features   

**Figure 2:**
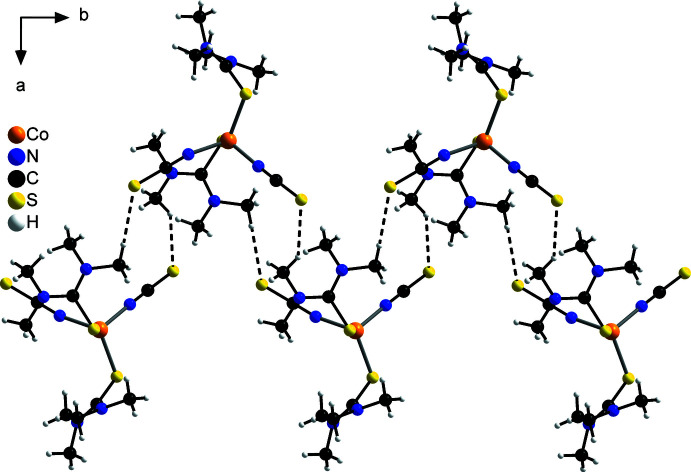
Crystal structure of the title compound with a view of the chains that run along the *b*-axis direction with inter­molecular C—H⋯S hydrogen bonds shown as dashed lines.

**Figure 3:**
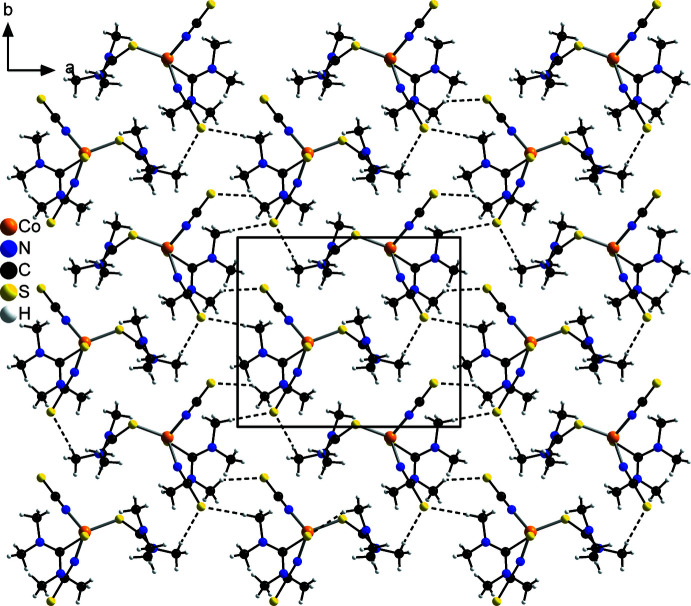
Crystal structure of the title compound with a view along the *c* axis of the layers. Inter­molecular C—H⋯S hydrogen bonds are shown as dashed lines.

**Figure 4:**
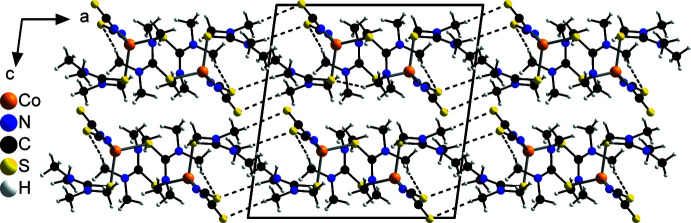
Crystal structure of the title compound with a view in the direction of the layers along the *b* axis. Inter­molecular C—H⋯S hydrogen bonds are shown as dashed lines.

As can be seen in Table 2 ▸, two sets of hydrogen bonds can be found for which the *D*—H⋯*A* angles are either relatively near to 180° (174.0 and 166.2°) or far from 180° (142.8 and 140.8°), which is indicative for strong or relatively weak hydrogen bonds, respectively. Although nearly all of these hydrogen bonds are inter­molecular bonds between the thio­cyanate sulfur and a C—H hydrogen atom from an adjacent complex, in one case relatively weak intra­molecular hydrogen C—H⋯S bonding between two different tetra­methyl­thio­urea mol­ecules of the same discrete complex is found. Each complex is connected to two different neighbouring complexes by pairs of C—H⋯S_NCS_ hydrogen bonds between the tetra­methyl­thio­urea coligands and the thio­cyanate anions. This leads to the formation of zigzag-like chains along the *b*-axis direction (Fig. 2 ▸), which are further connected by additional single C—H⋯S_NCS_ hydrogen bonds into layers that are parallel to the *ab* plane (Fig. 3 ▸). These layers are stacked along the *c*-axis direction with no pronounced inter­molecular inter­actions between them (Fig. 4 ▸).

## Database survey   

In the Cambridge Structural Database (Version 5.41, last update November 2019; Groom *et al.*, 2016 ▸) only 72 compounds containing transition-metal cations and tetra­methyl­thio­urea are reported, but none of them contains thio­cyanate anions. This search also reveals that no tetra­hedral Co(NCS)_2_ compounds with other thio­urea derivatives are known, but one chain compound with the composition [Co(NCS)_2_(thio­urea)_2_]_*n*_ has been reported (Rajarajan *et al.*, 2012 ▸). However, several structures built up of discrete tetra­hedral complexes with cobalt thio­cyanate and a variety of N-containing ligands are reported in the CCDC. These include, for example, bis­(3-methyl­pyridine)­diiso­thio­cyanato­cobalt(II) (Böckmann *et al.* 2011 ▸) and bis­(quinoline)­diiso­thio­cyanato­cobalt(II) (Mirčeva & Golič, 1990 ▸). It is noted that, in several cases, pyridine or imidazole derivatives are used that have large substituents adjacent to the coordinating N atoms, which might enforce the formation of a tetra­hedral complex for steric reasons.

## Synthesis and crystallization   

**General**

Co(NCS)_2_ and tetra­methyl­thio­urea were purchased from Sigma Aldrich and were used without further purification.

**Synthesis**

A suspension of Co(NCS)_2_ (0.50 mmol, 87.5 mg) and tetra­methyl­thio­urea (1.00 mmol, 132.23 mg) in 0.75 mL water was stored at 281 K. After a few days, deep-blue-coloured crystals were obtained, which were filtered off, and ground into powder or used for single-crystal structure determination. It is noted that no crystalline product could be obtained from an analogous reaction at room temperature. Elemental analysis calculated for C_12_H_24_N_6_CoS_4_ (439.56 g mol^−1^) C 32.79, H 5.50, N 19.12%, S 29.18, found: C 32.37, H 5.38, N 18.75, S 28.64.

**Experimental details**

Elemental analysis was performed using an EURO EA elemental analyser fabricated by EURO VECTOR Instruments.

The IR spectrum was measured using an ATI Mattson Genesis Series FTIR Spectrometer, control software: *WINFIRST*, from ATI Mattson.

The PXRD measurement was performed with Cu *K*α_1_ radiation (λ = 1.540598 Å) using a Stoe Transmission Powder Diffraction System (STADI P) equipped with a MYTHEN 1K detector and a Johansson-type Ge(111) monochromator.

DTA–TG measurements were performed in a dynamic nitro­gen atmosphere (100 sccm) in Al_2_O_3_ crucibles using a STA-PT 1600 thermobalance from Linseis. The instrument was calibrated using standard reference materials.

The DSC measurements were performed with a DSC 1 Star System with *STARe Excellence Software* from Mettler-Toledo AG. The instrument was calibrated using standard reference materials.

Thermomicroscopic measurements were performed using a hot-stage FP82 from Mettler and a BX60 microscope from Olympus, using the software analysis package from Mettler.

## Refinement   

Crystal data, data collection and structure refinement details are summarized in Table 3 ▸. All non-hydrogen atoms were refined anisotropically. The C—H atoms were positioned with idealized geometry (allowed to rotate but not to tip) and refined isotropically with *U*_iso_(H) = 1.5*U*_eq_(C).

## Supplementary Material

Crystal structure: contains datablock(s) I. DOI: 10.1107/S205698902001021X/tx2029sup1.cif

Structure factors: contains datablock(s) I. DOI: 10.1107/S205698902001021X/tx2029Isup2.hkl

Figure S1. IR spectrum of the title compound. DOI: 10.1107/S205698902001021X/tx2029sup3.tif

Figure S2. Experimental (top) and calculated (bottom) PXRD pattern of the title compound measured with Cu-radiation. DOI: 10.1107/S205698902001021X/tx2029sup4.tif

Figure S3. DTG, TG and DTA curve of the title compound measured at a rate of 4 degrees C/min. in a nitrogen atmosphere. DOI: 10.1107/S205698902001021X/tx2029sup5.tif

Figure S4. DSC heating and cooling curve of the title compound until the first endothermic event using a heating rate of 4 degrees C/min. under nitrogen atmosphere. DOI: 10.1107/S205698902001021X/tx2029sup6.tif

Figure S5. DSC heating and cooling curve of the title compound until the second endothermic event using a heating rate of 4 degrees C/min. under nitrogen atmosphere. DOI: 10.1107/S205698902001021X/tx2029sup7.tif

Figure S6. Thermomicroscopic images of the title compound at different temperatures when heated in air with 10 degrees C/min. DOI: 10.1107/S205698902001021X/tx2029sup8.tif

CCDC reference: 2018676

Additional supporting information:  crystallographic information; 3D view; checkCIF report

## Figures and Tables

**Table 1:** Selected geometric parameters (Å, °)

Co1—N1	1.9484 (17)	Co1—S11	2.3157 (5)
Co1—N2	1.9499 (17)	Co1—S21	2.3196 (6)
			
N1—Co1—N2	106.56 (7)	N1—Co1—S21	117.66 (5)
N1—Co1—S11	102.86 (5)	N2—Co1—S21	97.67 (6)
N2—Co1—S11	121.40 (5)	S11—Co1—S21	111.564 (19)

**Table 2:** Hydrogen-bond geometry (Å, °)

*D*—H⋯*A*	*D*—H	H⋯*A*	*D*⋯*A*	*D*—H⋯*A*
C12—H12*B*⋯S1^i^	0.98	2.93	3.906 (2)	174
C14—H14*A*⋯S2^ii^	0.98	2.93	3.741 (2)	141
C22—H22*C*⋯S11	0.98	2.61	3.442 (2)	143
C24—H24*A*⋯S1^iii^	0.98	2.92	3.878 (2)	166

Symmetry codes: (i) 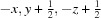; (ii) 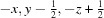; (iii) 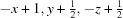.

**Table 3:** Experimental details

Crystal data	
Chemical formula	[Co(NCS)_2_(C_5_H_12_N_2_S)_2_]
*M*_r_	439.54
Crystal system, space group	Monoclinic, *P*2_1_/*c*
Temperature (K)	200
*a*, *b*, *c* (Å)	13.3288 (13), 11.2140 (8), 13.8579 (13)
β (°)	97.667 (8)
*V* (Å^3^)	2052.8 (3)
*Z*	4
Radiation type	Mo *K*α
μ (mm^−1^)	1.25
Crystal size (mm)	0.19 × 0.15 × 0.10

Data collection	
Diffractometer	Stoe IPDS2
Absorption correction	Numerical (*X-RED* and *X-SHAPE*; Stoe & Cie, 2002 ▸)
*T*_min_, *T*_max_	0.644, 0.805
No. of measured, independent and observed [*I* > 2σ(*I*)] reflections	14562, 4409, 3826
*R*_int_	0.044
(sin θ/λ)_max_ (Å^−1^)	0.639

Refinement	
*R*[*F* ^2^ > 2σ(*F* ^2^)], *wR*(*F* ^2^), *S*	0.031, 0.084, 1.02
No. of reflections	4409
No. of parameters	217
H-atom treatment	H-atom parameters constrained
Δρ_max_, Δρ_min_ (e Å^−3^)	0.33, −0.39

Computer programs: *X-AREA* (Stoe & Cie, 2002 ▸), *SHELXS97* and *XP* (Sheldrick, 2008 ▸), *SHELXL2018/3* (Sheldrick, 2015 ▸), *DIAMOND* (Brandenburg & Putz, 1999 ▸) and *publCIF* (Westrip, 2010 ▸).

## supplementary crystallographic information

### Crystal data

**Table:**

[Co(NCS)_2_(C_5_H_12_N_2_S)_2_]	*F*(000) = 916
*M**_r_* = 439.54	*D*_x_ = 1.422 Mg m^−^^3^
Monoclinic, *P*2_1_/*c*	Mo *K*α radiation, λ = 0.71073 Å
*a* = 13.3288 (13) Å	Cell parameters from 14562 reflections
*b* = 11.2140 (8) Å	θ = 2.4–27.0°
*c* = 13.8579 (13) Å	µ = 1.25 mm^−^^1^
β = 97.667 (8)°	*T* = 200 K
*V* = 2052.8 (3) Å^3^	Block, blue
*Z* = 4	0.19 × 0.15 × 0.10 mm

### Data collection

**Table:**

Stoe IPDS-2 diffractometer	3826 reflections with *I* > 2σ(*I*)
ω scans	*R*_int_ = 0.044
Absorption correction: numerical (X-Red and X-Shape; Stoe & Cie, 2002)	θ_max_ = 27.0°, θ_min_ = 2.4°
*T*_min_ = 0.644, *T*_max_ = 0.805	*h* = −15→16
14562 measured reflections	*k* = −13→14
4409 independent reflections	*l* = −17→17

### Refinement

**Table:**

Refinement on *F*^2^	Hydrogen site location: inferred from neighbouring sites
Least-squares matrix: full	H-atom parameters constrained
*R*[*F*^2^ > 2σ(*F*^2^)] = 0.031	*w* = 1/[σ^2^(*F*_o_^2^) + (0.0593*P*)^2^] where *P* = (*F*_o_^2^ + 2*F*_c_^2^)/3
*wR*(*F*^2^) = 0.084	(Δ/σ)_max_ = 0.001
*S* = 1.02	Δρ_max_ = 0.33 e Å^−^^3^
4409 reflections	Δρ_min_ = −0.39 e Å^−^^3^
217 parameters	Extinction correction: SHELXL2018/3 (Sheldrick 2018), Fc^*^=kFc[1+0.001xFc^2^λ^3^/sin(2θ)]^-1/4^
0 restraints	Extinction coefficient: 0.0092 (16)

### Special details

**Table:**

Geometry. All esds (except the esd in the dihedral angle between two l.s. planes) are estimated using the full covariance matrix. The cell esds are taken into account individually in the estimation of esds in distances, angles and torsion angles; correlations between esds in cell parameters are only used when they are defined by crystal symmetry. An approximate (isotropic) treatment of cell esds is used for estimating esds involving l.s. planes.

### Fractional atomic coordinates and isotropic or equivalent isotropic displacement parameters (Å^2^)

**Table:**

	*x*	*y*	*z*	*U*_iso_*/*U*_eq_	
Co1	0.31466 (2)	0.44406 (2)	0.18631 (2)	0.03623 (10)	
N1	0.26892 (14)	0.28853 (15)	0.13540 (11)	0.0473 (4)	
C1	0.22426 (14)	0.20050 (17)	0.11758 (12)	0.0400 (4)	
S1	0.16309 (4)	0.07801 (5)	0.09170 (4)	0.05239 (14)	
N2	0.23001 (14)	0.56237 (15)	0.11124 (12)	0.0476 (4)	
C2	0.18324 (14)	0.63196 (16)	0.06226 (12)	0.0389 (4)	
S2	0.11944 (5)	0.73069 (6)	−0.00388 (4)	0.05992 (16)	
S11	0.31339 (4)	0.42243 (5)	0.35233 (3)	0.04323 (13)	
C11	0.20254 (13)	0.33900 (15)	0.34598 (11)	0.0346 (3)	
N11	0.11381 (12)	0.39071 (14)	0.31533 (11)	0.0410 (3)	
C12	0.09967 (18)	0.51955 (19)	0.32179 (17)	0.0551 (5)	
H12A	0.150522	0.552429	0.372410	0.083*	
H12B	0.031805	0.536217	0.338259	0.083*	
H12C	0.107221	0.556414	0.259049	0.083*	
C13	0.02995 (16)	0.3274 (2)	0.25917 (16)	0.0580 (5)	
H13A	0.053452	0.249499	0.239227	0.087*	
H13B	0.004558	0.374051	0.201245	0.087*	
H13C	−0.024519	0.316034	0.299316	0.087*	
N12	0.20637 (12)	0.22456 (14)	0.37071 (10)	0.0392 (3)	
C14	0.12200 (18)	0.1612 (2)	0.40544 (16)	0.0538 (5)	
H14A	0.071235	0.218979	0.420747	0.081*	
H14B	0.147061	0.115724	0.464096	0.081*	
H14C	0.091237	0.106630	0.354706	0.081*	
C15	0.29981 (18)	0.1561 (2)	0.37811 (16)	0.0561 (5)	
H15A	0.345116	0.191657	0.335874	0.084*	
H15B	0.284447	0.073696	0.357751	0.084*	
H15C	0.332793	0.156901	0.445679	0.084*	
S21	0.47009 (4)	0.51223 (5)	0.15128 (3)	0.04435 (13)	
C21	0.56218 (13)	0.43472 (14)	0.22564 (11)	0.0333 (3)	
N21	0.57661 (12)	0.45344 (13)	0.32191 (10)	0.0369 (3)	
C22	0.54437 (16)	0.56303 (18)	0.36544 (14)	0.0463 (4)	
H22A	0.540529	0.627600	0.317434	0.069*	
H22B	0.593316	0.584178	0.422032	0.069*	
H22C	0.477595	0.550936	0.386096	0.069*	
C23	0.60727 (17)	0.3590 (2)	0.39199 (12)	0.0507 (5)	
H23A	0.610361	0.283021	0.357553	0.076*	
H23B	0.557930	0.352888	0.438242	0.076*	
H23C	0.674128	0.377561	0.427288	0.076*	
N22	0.62202 (12)	0.35804 (13)	0.18656 (9)	0.0384 (3)	
C24	0.72499 (16)	0.3301 (2)	0.23060 (14)	0.0509 (5)	
H24A	0.745874	0.386147	0.283738	0.076*	
H24B	0.771017	0.336746	0.181307	0.076*	
H24C	0.727201	0.248577	0.256314	0.076*	
C25	0.59760 (19)	0.3148 (2)	0.08646 (13)	0.0536 (5)	
H25A	0.523941	0.309911	0.069608	0.080*	
H25B	0.627351	0.235605	0.080900	0.080*	
H25C	0.625178	0.370024	0.041890	0.080*	

### Atomic displacement parameters (Å^2^)

**Table:**

	*U*^11^	*U*^22^	*U*^33^	*U*^12^	*U*^13^	*U*^23^
Co1	0.03421 (15)	0.03936 (14)	0.03465 (14)	0.00045 (9)	0.00284 (9)	0.00153 (8)
N1	0.0544 (10)	0.0439 (9)	0.0433 (8)	−0.0005 (7)	0.0056 (7)	−0.0033 (6)
C1	0.0435 (10)	0.0433 (9)	0.0341 (8)	0.0064 (8)	0.0078 (7)	0.0016 (6)
S1	0.0532 (3)	0.0467 (3)	0.0576 (3)	−0.0074 (2)	0.0087 (2)	−0.0008 (2)
N2	0.0442 (9)	0.0477 (9)	0.0489 (9)	0.0033 (7)	−0.0017 (7)	0.0035 (7)
C2	0.0350 (9)	0.0444 (9)	0.0374 (8)	−0.0011 (7)	0.0049 (6)	−0.0005 (7)
S2	0.0516 (3)	0.0689 (4)	0.0587 (3)	0.0139 (3)	0.0055 (2)	0.0237 (3)
S11	0.0358 (3)	0.0584 (3)	0.0352 (2)	−0.00976 (19)	0.00353 (16)	−0.00086 (17)
C11	0.0327 (8)	0.0426 (8)	0.0288 (6)	0.0009 (7)	0.0053 (6)	−0.0055 (6)
N11	0.0335 (8)	0.0444 (8)	0.0446 (7)	0.0057 (6)	0.0036 (6)	−0.0023 (6)
C12	0.0537 (13)	0.0477 (11)	0.0659 (12)	0.0154 (9)	0.0155 (10)	0.0021 (9)
C13	0.0371 (11)	0.0755 (15)	0.0574 (11)	0.0002 (10)	−0.0080 (8)	−0.0021 (10)
N12	0.0382 (8)	0.0401 (8)	0.0395 (7)	0.0033 (6)	0.0056 (6)	−0.0009 (6)
C14	0.0545 (13)	0.0496 (11)	0.0571 (11)	−0.0128 (9)	0.0068 (9)	0.0030 (9)
C15	0.0566 (13)	0.0549 (12)	0.0573 (11)	0.0209 (10)	0.0096 (9)	0.0043 (9)
S21	0.0363 (3)	0.0573 (3)	0.0390 (2)	−0.00071 (19)	0.00352 (16)	0.01763 (18)
C21	0.0341 (9)	0.0352 (8)	0.0307 (7)	−0.0069 (6)	0.0048 (6)	0.0035 (6)
N21	0.0404 (8)	0.0407 (7)	0.0295 (6)	0.0014 (6)	0.0049 (5)	0.0007 (5)
C22	0.0430 (11)	0.0498 (10)	0.0477 (9)	−0.0052 (8)	0.0118 (8)	−0.0134 (8)
C23	0.0588 (13)	0.0611 (12)	0.0320 (8)	0.0063 (10)	0.0048 (7)	0.0106 (8)
N22	0.0468 (9)	0.0381 (7)	0.0303 (6)	−0.0006 (6)	0.0055 (5)	−0.0012 (5)
C24	0.0517 (12)	0.0556 (11)	0.0458 (9)	0.0138 (9)	0.0083 (8)	0.0007 (8)
C25	0.0690 (14)	0.0571 (11)	0.0355 (9)	−0.0105 (10)	0.0107 (8)	−0.0100 (8)

### Geometric parameters (Å, º)

**Table:**

Co1—N1	1.9484 (17)	N11—C13	1.459 (3)
Co1—N2	1.9499 (17)	N11—C12	1.461 (3)
Co1—S11	2.3157 (5)	N12—C15	1.455 (3)
Co1—S21	2.3196 (6)	N12—C14	1.465 (3)
N1—C1	1.162 (3)	S21—C21	1.7282 (17)
C1—S1	1.613 (2)	C21—N22	1.336 (2)
N2—C2	1.160 (2)	C21—N21	1.339 (2)
C2—S2	1.6066 (18)	N21—C23	1.458 (2)
S11—C11	1.7411 (18)	N21—C22	1.459 (2)
C11—N12	1.327 (2)	N22—C24	1.460 (3)
C11—N11	1.335 (2)	N22—C25	1.464 (2)
			
N1—Co1—N2	106.56 (7)	C11—N11—C12	121.74 (17)
N1—Co1—S11	102.86 (5)	C13—N11—C12	114.72 (17)
N2—Co1—S11	121.40 (5)	C11—N12—C15	122.09 (17)
N1—Co1—S21	117.66 (5)	C11—N12—C14	123.28 (16)
N2—Co1—S21	97.67 (6)	C15—N12—C14	114.12 (17)
S11—Co1—S21	111.564 (19)	C21—S21—Co1	107.02 (6)
C1—N1—Co1	164.57 (16)	N22—C21—N21	119.39 (15)
N1—C1—S1	179.27 (18)	N22—C21—S21	119.81 (12)
C2—N2—Co1	175.96 (17)	N21—C21—S21	120.76 (13)
N2—C2—S2	178.70 (18)	C21—N21—C23	122.68 (15)
C11—S11—Co1	97.16 (5)	C21—N21—C22	122.21 (15)
N12—C11—N11	120.27 (16)	C23—N21—C22	114.11 (15)
N12—C11—S11	120.22 (13)	C21—N22—C24	123.21 (14)
N11—C11—S11	119.51 (14)	C21—N22—C25	121.86 (16)
C11—N11—C13	122.80 (17)	C24—N22—C25	113.75 (16)

### Hydrogen-bond geometry (Å, º)

**Table:**

*D*—H···*A*	*D*—H	H···*A*	*D*···*A*	*D*—H···*A*
C12—H12*B*···S1^i^	0.98	2.93	3.906 (2)	174
C14—H14*A*···S2^ii^	0.98	2.93	3.741 (2)	141
C22—H22*C*···S11	0.98	2.61	3.442 (2)	143
C24—H24*A*···S1^iii^	0.98	2.92	3.878 (2)	166

Symmetry codes: (i) −*x*, *y*+1/2, −*z*+1/2; (ii) −*x*, *y*−1/2, −*z*+1/2; (iii) −*x*+1, *y*+1/2, −*z*+1/2.

## References

[bb1] Abedi, M., Kirschbaum, K., Shamkhali, A. N., Brue, C. R. & Khandar, A. A. (2016). *Polyhedron*, **109**, 176–181.

[bb2] Barnett, S. A., Blake, A. J., Champness, N. R. & Wilson, C. (2002). *Chem. Commun.* pp. 1640–1641. 10.1039/b203661d 12170821

[bb3] Bhowmik, P., Chattopadhyay, S., Drew, M. G. B., Diaz, C. & Ghosh, A. (2010). *Polyhedron*, **29**, 2637–2642.

[bb4] Böckmann, J., Reimer, B. & Näther, C. (2011). *Z. Naturforsch. B: Chem. Sci.* **66**, 819–827.

[bb5] Böhme, M., Jochim, A., Rams, M., Lohmiller, T., Suckert, S., Schnegg, A., Plass, W. & Näther, C. (2020). *Inorg. Chem.* **59**, 5325–5338. 10.1021/acs.inorgchem.9b03357 32091883

[bb6] Brandenburg, K. & Putz, H. (1999). *DIAMOND*. Crystal Impact GbR, Bonn, Germany.

[bb7] Buckingham, S. (1994). *Coord. Chem. Rev.* **135–136**, 587–621.

[bb8] Devillanova, F. A. (2007). Editor. *Handbook of Chalcogen Chemistry: New Perspectives in Sulfur, Selenium and Tellurium, 1st* ed., pp. 107–108. Cambridge: Royal Society of Chemistry.

[bb9] Groom, C. R., Bruno, I. J., Lightfoot, M. P. & Ward, S. C. (2016). *Acta Cryst.* B**72**, 171–179. 10.1107/S2052520616003954 PMC4822653 27048719

[bb10] Haasnoot, J. G., Driessen, W. L. & Reedijk, J. (1984). *Inorg. Chem.* **23**, 2803–2807.

[bb11] Jin, Y., Che, Y. X. & Zheng, J. M. (2007). *J. Coord. Chem.* **60**, 2067–2074.

[bb12] Jochim, A., Lohmiller, T., Rams, M., Böhme, M., Ceglarska, M., Schnegg, A., Plass, W. & Näther, C. (2020). *Inorg. Chem.* **59**, 8971–8982. 10.1021/acs.inorgchem.0c00815 32551545

[bb13] Mautner, F. A., Traber, M., Fischer, R. C., Torvisco, A., Reichmann, K., Speed, S., Vicente, R. & Massoud, S. S. (2018). *Polyhedron*, **154**, 436–442.

[bb14] Mirčeva, A. & Golič, L. (1990). *Acta Cryst.* C**46**, 1001–1003.

[bb15] Neumann, T., Jess, I., Pielnhofer, F. & Näther, C. (2018*b*). *Eur. J. Inorg. Chem.* pp. 4972–4981.

[bb16] Neumann, T., Rams, M., Tomkowicz, Z., Jess, I. & Näther, C. (2019). *Chem. Commun.* **55**, 2652–2655. 10.1039/c8cc09392j 30742155

[bb17] Neumann, T., Rams, M., Wellm, C. & Näther, C. (2018*a*). *Cryst. Growth Des.* **18**, 6020–6027.

[bb18] Palion-Gazda, J., Machura, B., Lloret, F. & Julve, M. (2015). *Inorg. Chem.* **56**, 2380–2388. 10.1021/acs.inorgchem.7b00360 28530402

[bb19] Prananto, Y. P., Urbatsch, A., Moubaraki, B., Murray, K. S., Turner, D. R., Deacon, G. B. & Batten, S. R. (2017). *Aust. J. Chem.* **70**, 516–528.

[bb20] Rajarajan, K., Sendil Kumar, K., Ramesh, V., Shihabuddeen, V. & Murugavel, S. (2012). *Acta Cryst.* E**68**, m1125–m1126. 10.1107/S1600536812033193 PMC3414167 22904774

[bb21] Rams, M., Jochim, A., Böhme, M., Lohmiller, T., Ceglarska, M., Rams, M. M., Schnegg, A., Plass, W. & Näther, C. (2020). *Chem. Eur. J.* **26**, 2837–2851. 10.1002/chem.201903924 PMC7078958 31702081

[bb22] Rams, M., Tomkowicz, Z., Böhme, M., Plass, W., Suckert, S., Werner, J., Jess, I. & Näther, C. (2017). *Phys. Chem. Chem. Phys.* **19**, 3232–3243. 10.1039/c6cp08193b 28083584

[bb23] Robinson, K., Gibbs, G. V. & Ribbe, P. H. (1971). *Science*, **172**, 567–570. 10.1126/science.172.3983.567 17802221

[bb24] Sheldrick, G. M. (2008). *Acta Cryst.* A**64**, 112–122. 10.1107/S0108767307043930 18156677

[bb25] Sheldrick, G. M. (2015). *Acta Cryst.* C**71**, 3–8.

[bb26] Shurdha, E., Moore, C. E., Rheingold, A. L., Lapidus, S. H., Stephens, P. W., Arif, A. M. & Miller, J. S. (2013). *Inorg. Chem.* **52**, 10583–10594. 10.1021/ic401558f 23981238

[bb27] Stoe & Cie (2002). *X-AREA*, *X-RED* and *X-SHAPE*. Stoe & Cie, Darmstadt, Germany.

[bb28] Suckert, S., Rams, M., Böhme, M., Germann, L. S., Dinnebier, R., Plass, W., Werner, J. & Näther, C. (2016). *Dalton Trans.* **45**, 18190–18201. 10.1039/c6dt03752f 27796392

[bb29] Werner, J., Rams, M., Tomkowicz, Z., Runčevski, T., Dinnebier, R. E., Suckert, S. & Näther, C. (2015*a*). *Inorg. Chem.* **54**, 2893–2901. 10.1021/ic503029t 25741770

[bb30] Werner, S., Tomkowicz, Z., Rams, M., Ebbinghaus, S. G., Neumann, T. & Näther, C. (2015*b*). *Dalton Trans.* **44**, 14149–14158. 10.1039/c5dt01898f 26182402

[bb31] Westrip, S. P. (2010). *J. Appl. Cryst.* **43**, 920–925.

