# Supplementary figures and images for: Crystal structure of bis­(tetra­methyl­thio­urea-κS)bis(thio­cyanato-κN)cobalt(II)
Source: Acta Crystallogr E Crystallogr Commun. 2020 Jul 31;76(Pt 8):1373–7. doi: 10.1107/S205698902001021X (PMC7405571; doi:10.1107/S205698902001021X)

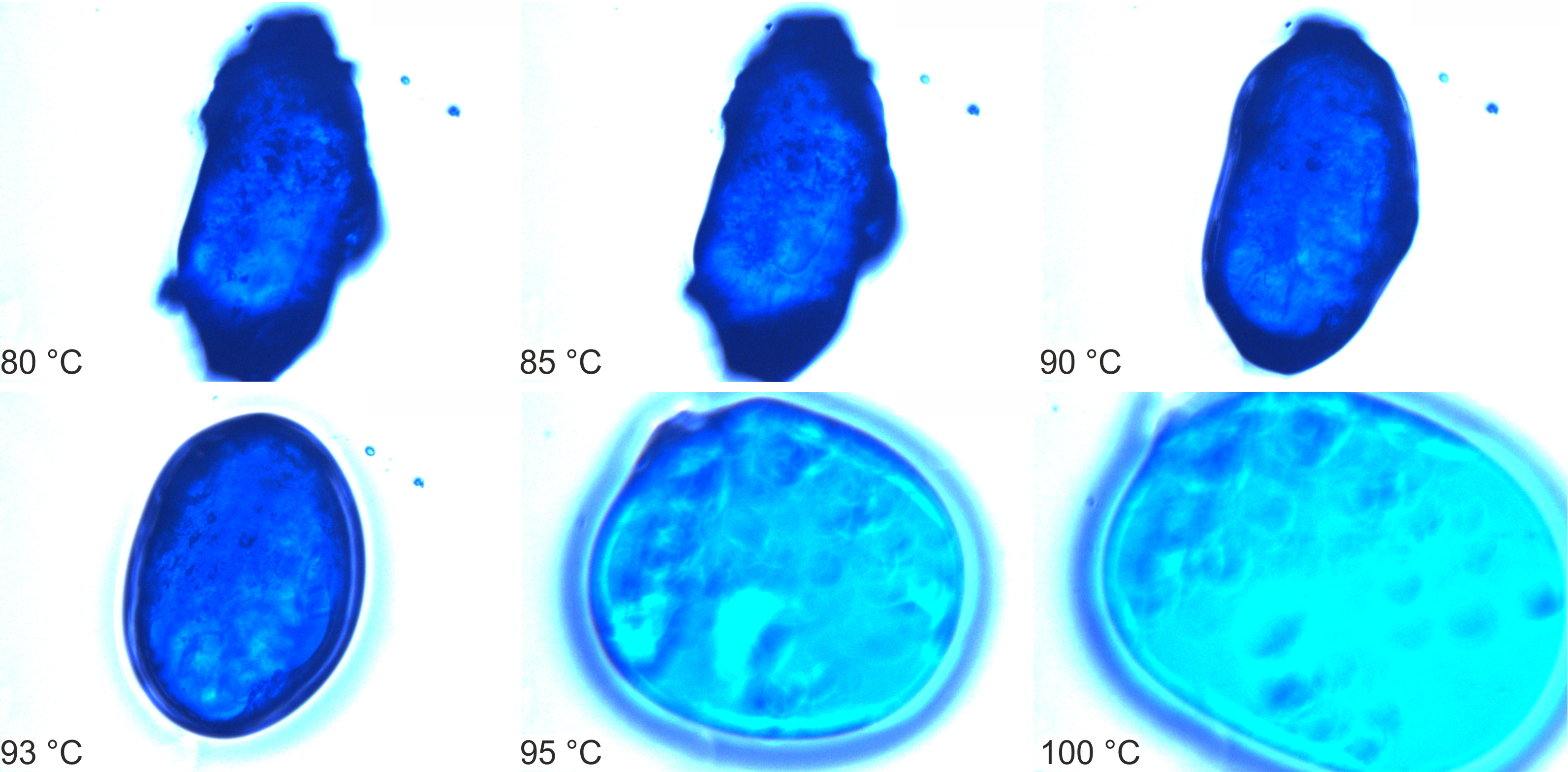

Supplement: Supplementary file 8 [file e-76-01373-sup8.tif]
